# Supplementary material for: End Sequence Analysis Toolkit (ESAT) expands the extractable information from single-cell RNA-seq data
Source: Genome Res. 2016 Oct;26(10):1397–410. doi: 10.1101/gr.207902.116 (PMC5052061; doi:10.1101/gr.207902.116)
Supplement: Supplemental Material [file supp_gr.207902.116_Supp_Materials.tar.gz › ESAT-esat_v0.1/doc/html/classumms_1_1core_1_1scripture_1_1statistics_1_1_connect_disconnected_transcripts.html]

ESAT: umms.core.scripture.statistics.ConnectDisconnectedTranscripts Class Reference


ESAT


- Main Page
- Related Pages
- Packages
- Classes
- Files

- Class List
- Class Index
- Class Hierarchy
- Class Members

All Classes Namespaces Files Functions Variables Enumerator Pages

- umms
- core
- scripture
- statistics
- ConnectDisconnectedTranscripts

Public Member Functions |
Static Public Member Functions |
List of all members

umms.core.scripture.statistics.ConnectDisconnectedTranscripts Class Reference

Collaboration diagram for umms.core.scripture.statistics.ConnectDisconnectedTranscripts:

[legend]

|  |  |
| --- | --- |
| Public Member Functions | |
|  | ConnectDisconnectedTranscripts (String annotationFile, String outputName, File bamFile, TranscriptionRead strand, int maxSize) throws IOException |
|  | |
| void | doWork (String outputName) throws IOException |
|  | |

|  |  |
| --- | --- |
| Static Public Member Functions | |
| static Pair< Gene > | getOrderedAssembly (Gene gene1, Gene gene2) |
|  | |
| static void | main (String[] args) throws ParseException, IOException |
|  | |

## Constructor & Destructor Documentation

|  |  |  |  |
| --- | --- | --- | --- |
| umms.core.scripture.statistics.ConnectDisconnectedTranscripts.ConnectDisconnectedTranscripts | ( | String | *annotationFile*, |
|  |  | String | *outputName*, |
|  |  | File | *bamFile*, |
|  |  | TranscriptionRead | *strand*, |
|  |  | int | *maxSize* |
|  | ) |  | throws IOException |

Here is the call graph for this function:

Here is the caller graph for this function:

## Member Function Documentation

|  |  |  |  |  |  |
| --- | --- | --- | --- | --- | --- |
| void umms.core.scripture.statistics.ConnectDisconnectedTranscripts.doWork | ( | String | *outputName* | ) | throws IOException |

Here is the call graph for this function:

Here is the caller graph for this function:

|  |  |  |  |
| --- | --- | --- | --- |
| static Pair<Gene> umms.core.scripture.statistics.ConnectDisconnectedTranscripts.getOrderedAssembly | ( | Gene | *gene1*, |
|  |  | Gene | *gene2* |
|  | ) |  |  |

static

Here is the call graph for this function:

|  |  |  |  |  |  |
| --- | --- | --- | --- | --- | --- |
| static void umms.core.scripture.statistics.ConnectDisconnectedTranscripts.main | ( | String[] | *args* | ) | throws ParseException, IOException |

static

Here is the call graph for this function:

---

The documentation for this class was generated from the following file:

- java/umms/core/scripture/statistics/ConnectDisconnectedTranscripts.java


---

Generated on Fri Aug 15 2014 15:11:15 for ESAT by  

 1.8.7
